# Voice and Temporal Auditory Processing in Elderly People: A Correlation Study

**DOI:** 10.1055/s-0043-1768139

**Published:** 2024-02-05

**Authors:** Mariana Batista de Souza Santos, Lílian Ferreira Muniz, Adriana de Oliveira Camargo Gomes, Cleide Fernandes Teixeira, Karina Paes Advíncula, Zulina Souza de Lira, Bruno Teixeira de Moares, Jonia Alves Lucena

**Affiliations:** 1 Department of Speech Therapy, Centro de Ciências da Saúde, Universidade Federal de Pernambuco, Recife, Pernambuco, Brazil; 2 Otorhinolaryngology, Centro de Ciências da Saúde, Universidade Federal de Pernambuco, Recife, Pernambuco, Brazil

**Keywords:** speech, language, hearing, pathology, aging, voice, older adult

## Abstract

**Introduction** The voice and hearing can be affected to different degrees by aging, which can cause communication difficulties for elderly people. Vocal production requires effective temporal auditory processing at central levels within the nervous system, which can be compromised by the aging process.

**Objective** To analyze the correlation between voice and temporal auditory processing in older adults.

**Materials and Methods** A total of 40 elderly people aged 60 years or older were subdivided into 2 groups according to the presence or absence of vocal symptoms measured by the Voice Symptom Scale. All of the participants were submitted to auditory temporal tests, vocal self-assessment, and acoustic and perceptual auditory analyses of voice.

**Results** Most of the subjects assessed had decreased voice intensity and normal variability in terms of vocal quality. The performance was normal in the Pitch Pattern Sequence test and altered in the Random Gap Detection test. In the Masking Period Pattern test, the detection thresholds for the target signal were increased in the presence of masking in different temporal target signal positions. Only pitch differed between the two groups. There were differences between the genders regarding frequency, shimmer, the overall severity of the alteration, and roughness. There was a correlation regarding temporal resolution ability and the overall severity of the alteration and roughness of the voice.

**Conclusion** There is a central auditory impairment in temporal resolution which is correlated with vocal alterations in the elderly.

## Introduction

The quantitative assessment of auditory changes during the aging process supports the development of appropriate therapeutic objectives. Different biopsychosocial disorders also occur naturally during the aging process. In particular, as age advances, voice and hearing can be affected, causing communication difficulties. This can impact on social life, leading to the risk of specific consequences that prevent the person from successfully adjusting and adapting to aging. 1 2

The pathophysiological processes that affect the aging auditory system include sensorial alterations in hearing that affect the auditory nerve, the cochlea, and structures associated with the central temporal processing of the complex acoustic stimuli. 3 4 5 6 In the case of the voice, aging can affect not only the larynx and its neural and muscular modulations, but also associated systems, such as the respiratory capacity and coordination. The process of natural voice aging is called presbyphonia. 7 8 9

Auditory integrity at the peripheral and/or central levels is essential for adequate voice production. 10 Temporal auditory processing is related to the processing of the minimal acoustic events necessary to produce and perceive speech. It is an essential component in oral communication because it is associated with the distinction of sound features and the perception of frequency and intensity, such as it occurs regarding musical notes and scales. 11 12 It is believed that the association between auditory and vocal functions highlights the importance of considering auditory feedback in the voice assessment and therapy process in elderly people.

Therefore, the purpose of the present study was to analyze the relationship between the voice of elderly people and temporal auditory processing abilities.

## Materials and Methods

The present was a cross-sectional, analytical, and correlational study conducted in a referral center for elderly care at a public university in the state of Pernambuco, Brazil. The study was approved by the Ethics in Research Committee (under protocol number 148/234). All subjects consented to participate, and data collection took place between May and December 2018.

A total of 40 female and male adults aged 60 years or older were included in the study. The participants either had normal tonal hearing thresholds (≤ 25dB in the octave frequencies from 250 Hz to 8,000 Hz) or characteristics of presbycusis (bilateral, symmetric sensory hearing loss, with descending configuration, with an average among the thresholds of 500 Hz, 1,000 Hz and 2,000 Hz of up to 25 dB). Only subjects with no otorhinolaryngological report of the presbylarynx and no report of laryngeal disorders were included. Potential participants with a history of neurological or cognitive disorders that could affect the voice or hearing were excluded. Candidates with conductive or mixed hearing losses were also excluded, since these can be temporary, modifying hearing in a unpredictable manner.

Based on these inclusion and exclusion criteria, the participants were initially referred for an otorhinolaryngological evaluation to exclude possible laryngeal pathologies. In a later session, the subjects were assessed through screening and auditory characterization. The researcher performed a visual inspection of the external acoustic meatus, measured the tone auditory thresholds through audiometry, and evaluated the condition of the tympano-ossicular system by acoustic immittanciometry.

Tonal audiometry was performed by air conduction and, when there was any alteration in the hearing threshold, the tonal hearing thresholds were searched using bone conduction in order to characterize the type of hearing loss, discarding the possibility of conductive or mixed losses. Acoustic immitanciometry was used to assess the mobility conditions in the middle ear and the operating conditions of the tympano-ossicular system, ruling out changes in sound conduction. For that was performed, in which a pressure variation of +200 daPa to −200 daPa was emitted in the external acoustic meatus of the individual. Then, through this same probe and a contralateral earphone, pure tones were presented to carry out the investigation of the acoustic reflex thresholds.

Subsequently, to eliminate the possible bias of cognitive impairment, a cognitive assessment was also carried out using the Mini Mental State Examination (MMSE) adapted for use in Brazil. 13 In this protocol, the cutoff score is related to different levels of schooling, ranging from 20 to 28 points. Only the subjects who obtained a score above the cutoff point were included in the present study.

Finally, after choosing the participants, the assessment of vocal signs and symptoms was performed. The participants were asked about the presence of voice complaints, answering “yes” or “no” regarding the occurrence of throat clearing, vocal fatigue, hoarseness, unstable voice, thick voice, weak voice, thin voice, and effort to speak. The participants were also asked to self-assess their vocal quality. For this, they were instructed to classify their voices as *excellent* , *very good* , *good* , *reasonable* , and *bad* , assingning them scores of 0, 1, 2, 3 or 4 respectively. Such scores were also considered in the vocal evaluation of the participants.

The Voice Symptom Scale (VoiSS) was used because it has a high degree of validity, reliability, and responsiveness not only regarding the identification of signs and symptoms of vocal disorders, but also regarding the impact of the vocal disorder. The VoiSS has a cutoff score indicative of voice alteration, and it is frequently used as a screening tool; the cutoff score was set at 16 points. 14

The VoiSS protocol was applied to divide the subjects in two groups: group I – with voice symptoms (scores ≥ 16 points) and Group II – without voice symptoms (scores < 16 points). 14

In addition, a headset microphone (model HT-2, Enping Karsect Electronics Co., Ltd., Enping, Guangdong, China) and a noise-reducing card (PureAudio USB-SA digital audio adapter, Andrea Communications, Farmingdale, NY, United States) were attached to an Intel Core i3–2348M Notebook computer (Intel Corporation, Santa Clara, CA, United States). To minimize interference in the records, the microphone was kept at a distance of approximately 4 cm from the mouth. Each participant was asked to emit the sustained vowel /a/ and count from 1 to 10, which was recorded in the FonoView (CTS Informática, Pato Branco, PR, Brazil) software. The Participants were also asked to produce the vowel /ɛ/ in the Voxmetria software (CTS Informática). The following parameters were extracted using Voxmetria: fundamental frequency, intensity, jitter, shimmer, and the glottal-to-noise excitation ratio (GNE).

The voice samples were edited in the software, disregarding the beginning and end of the emission, which avoided the initial attack of the emission and use of reserve air at the end. The sampling range was of 44,100 Hz and quantization was performed at 16 bits.

The samples obtained from the FonoView software were categorised by three double-blinded voice specialists. Six vocal parameters were evaluated: overall severity of alteration (OSA), roughness, breathiness, tension, pitch, and loudness. 15 The judges listened to the participants' voices at random and performed the judgment, measuring the degree of voice deviation using the Visual Analogue Scale (VAS).

The numerical correspondences in the VAS parameters were categorized as: normal variability of voice quality or absence of deviation (0 to 35.5 mm); slight deviation (35.6 to 50.5 mm); moderate deviation (50.6 to 90.5 mm); and intense deviation (90.6 to 100 mm). 15 To analyze the reproducibility of the responses, as well as the internal reliability of the evaluators, 30% of the speech samples were randomly selected for repeated analysis by each of the judges.

To assess temporal auditory processing with regards to the temporal auditory abilities (ordering, resolution, and masking), the following tests were applied: Pitch Pattern Sequence (PPS), Random Gap Detection Test (RGDT), and Masking Period Pattern (MPP). 16

The The PPS test consisted of the monaural presentation of 10 non-verbal stimuli, at 50 dB SL, of 10 sequences combined in different ways. Each contained 3 stimulus tones that differed in frequency: 1,430 Hz for the high frequencies (H) and 880 Hz for the low frequencies (L). The test has six possibilities of combination: (HHL), (HLH), (HLL), (LHH), (LHL), and (HHL). The participants were instructed to name the stimuli, in the order of their presentation, as *low* and *high* or *thick* and *thin* . 17 Participants who achieved at least 75% of correct answers were considered normal.

In the RGDT, gaps in noise were presented at 50 dB SL above the tritonal average (500 Hz to 2,000 Hz) of the audiometric test. Pure-tone sound stimuli were presented binaurally at the frequencies of 500 Hz, 1,000 Hz, 2,000 Hz, and 4,000 Hz, according to the test protocol. 18 Each frequency consisted of nine paired presentations, and at each of these frequencies the presentation of the interstimulus intervals was randomized. The participant should say whether one or two tones was heard.

The interval between the tone presentations ranged from 0 ms to 40 ms, in random order, with increments ranging from 2 ms to 10 ms. In cases in which the subjects did not identify the random intervals until the interval reached 40 ms, the expanded version proposed by the test protocol was used. 18 For the analysis, we considered the shortest interval in which the individual could identify the presence of two tones for each frequency. The detection threshold of random intervals was calculated as the average of the four frequencies evaluated. The result was considered normal if it was up to 23,13 ms.

The stimuli of the MPP test were generated using a digital signal processing platform (RZ6, Tucker-Davis Technologies, Alachua, FL, United States), together with a customized MATLAB (MathWorks, Natick, MA, United States) script. They were presented to the right ear or to the best ear, when the thresholds obtained between the ears varied more than 5 dB, using a headphone (HD580, Sennheiser, Wedemark, Germany). There is no normative standard to apply the MPP to the elderly population; it is still in testing phase.

To assess temporal masking using the MPP test, it is necessary to collect auditory thresholds before starting for the target tone with steady-state noise ate 65 dB SPL (Steady-high) and 30 dB SPL (Steady-low). To apply the test, the masking noise was presented at an intensity of 65 dB SPL, for 400 ms, followed by an abrupt decrease to an intensity of 30 dB SPL, remaining thus for 400 ms; then, the noise was increased again to 65 dB SPL. This presentation sequence provoked the auditory sensation of 3 independent noises, with the presentation of the target signal in 3 random time intervals (35 ms, 135 ms, and 200 ms). The subjects were instructed to pay attention to three sounds and to use a handheld box with three light buttons to identify the sound that differed from the others. Then, the average was calculated to determine the lowest intensity of perception of the target signal for each time interval investigated. These time intervals refer to the target signal presentation interval after the start of the masking noise modulation.

The Statistical Package for the Social Sciences (SPSS Statistics for Windows, SPSS Inc., Chicago, IL, United States), version 17.1, was used for the descriptive and statistical analysis of the data. The Shapiro-Wilk test was applied to verify the hypothesis of normality, and the Levene F test, to verify the equality of the variances. The Student *t* -test was used for variables with normal distribution in each of the groups, and the Mann-Whitney and Spearman correlation tests were used in cases of rejection of normality in at least one of the groups or between one of the variables.

The Pearson Chi-squared test or the Fisher exact test were applied to compare the groups in terms of the categorical variables or to assess the association between two categorical variables. The Student *t* -test with equal variances or the Mann-Whitney test were used to compare the groups regarding the numerical variables or to determine the association between two categories. To assess the degree of correlation between two numerical variables, the Spearman correlation coefficient was used.

The speech samples for the perceptual auditory analysis were submitted to a reproducibility analysis using the Kappa coefficient (k) values. The Kappa score is a measure that varies between −1 and +1 and, when equal to 1, it indicates perfect agreement; an index of zero suggests agreement equivalent to chance or independence between assessments or between examiners. From this analysis, the evaluator with the highest agreement in the responses, that is, the highest weighted Kappa index, was selected.

The margin of error used in deciding the significance of the statistical tests was of 5% ( *p* ≤ 0.05).

## Results

The average age of the participants was of 67.63 years. Gender distributiin was equal: 20 men and 20 women. The total and subscale scores on the VoiSS are shown in Table 1 , and the functional and physical scores were significantly higher in group I.

**Table 1:** Scores of the study groups on the Voice Symptom Scale

	Study groups		
Variable	Group I: mean ± standard deviation (median)	Group II: nean ± standard deviation (median)	*p* -value
TOTAL	n = 20	n = 20	
Total score	32.20 ± 2.89 (33.0)	9.95 ± 1.05 (9.50)	*p*^a^ < 0.001*
Functional score	17.90 ± 1.87 (17.50)	5.25 ± 0.66 (6.00)	*p*^a^ < 0.001*
Emotional score	5.80 ± 1.02 (5.50)	3.55 ± 0.55 (3.00)	*p*^a^ = 0.074
Physical score	8.50 ± 1.33 (8.50)	1.15 ± 0.293 (1.00)	*p*^a^ < 0.001*

Notes: *Significant difference at the level of 5.0%. ^a^ Mann-Whitney test.

The voice complaints reported are presented in Table 2 . Except for deep voice, the percentages referring to each complaint were higher among the subjects who had scored above the cutoff in the VoiSS (group I). The most common voice complaints, throat clearing, fatigue when speaking, and weak voice, were more common in Group I.

**Table 2:** Voice complaints or issues reported by the study groups

	Study groups						
Variable	Group I		Group II		Whole sample		*p* -value
	n	%	n	%	N	%	
TOTAL	20	100.0	20	100.0	40	100.0	
**Voice complaints/issues**							
Yes	18	90.0	18	90.0	36	90.0	*p*^a^ = 1.000
No	2	10.0	2	10.0	4	10.0	
***Type of voice complaint/issue***							
**Phlegm**							
Yes	14	70.0	7	35.0	21	52.5	*p*^b^ = 0.027*
No	6	30.0	13	65.0	19	47.5	
**Fatigue when speaking**							
Yes	13	65.0	6	30.0	19	47.5	*p*^b^ = 0.027*
No	7	35.0	14	70.0	21	52.5	
**Hoarseness**							
Yes	8	40.0	4	20.0	12	30.0	*p*^b^ = 0.168
No	12	60.0	16	80.0	28	70.0	
**Unstable voice**							
Yes	7	35.0	5	25.0	12	30.0	*p*^b^ = 0.490
No	13	65.0	15	75.0	28	70.0	
**Deep voice**							
Yes	4	20.0	6	30.0	10	25.0	*p*^b^ = 0.465
No	16	80.0	14	70.0	30	75.0	
**Weak voice**							
Yes	8	40.0	1	5.0	9	22.5	*p*^a^ = 0.020*
No	12	60.0	19	95.0	31	77.5	
**Shrill voice**							
Yes	5	25.0	3	15.0	8	20.0	*p*^a^ = 0.695
No	15	75.0	17	85.0	32	80.0	
**Effort to speak**							
Yes	6	30.0	2	10.0	8	20.0	*p*^a^ = 0.235
No	14	70.0	18	90.0	32	80.0	
***Self-evaluation of voice***							
Excellent/Very good/Good	9	45.0	15	75.0	24	60.0	*p*^b^ = 0.053
Not so good/Bad	11	55.0	5	25.0	16	40.0	

Notes: *Significant difference at the level of 5.0%. ^a^ Fisher exact test. ^b^ Pearson Chi-squared test.

The self-evaluation of voice quality ( Table 2 ) also shows that most of the participants in group I reported a negative impact (not so good or bad). However, considering the totality of adults assessed, most evaluated their voice positively, as excellent, very good or good.

Group comparisons regarding the acoustic parameters of the voices are presented by gender in Table 3 . Only the fundamental frequency of the voice differed between men and women. No differences were found between subjects with and without voice symptoms. Vocal intensity was diminished in both groups.

**Table 3:** Mean values of the acoustic analysis mean values by study group

	Study groups			
	Group I (n = 20)	Group II (n = 20)	Whole sample (N = 40)	
Variable	Mean ± standard deviation (median)	Mean ± standard deviation (median)	Mean ± standard deviation (median)	*p* -value
**Acoustic parameters**				
Fundamental frequency (male)	120.85 ± 17.58 (120.30)	113.44 ± 12.15 (110.84)	117.15 ± 15.19 (116.48)	*p*^a^ = 0.287
Fundamental frequency (female)	183.63 ± 22.70 (182.05)	171.26 ± 19.78 (172.53)	177.45 ± 21.67 (179.29)	*p*^b^ = 0.326
Intensity	57.48 ± 6.42 (57.87)	58.61 ± 5.19 (58.99)	58.05 ± 5.79 (58.32)	*p*^a^ = 0.543
Jitter	0.74 ± 1.19 (0.28)	0.37 ± 0.30 (0.28)	0.55 ± 0.88 (0.28)	*p*^b^ = 0.787
Shimmer	6.51 ± 4.54 (5.01)	5.09 ± 3.24 (3.74)	5.80 ± 3.96 (4.30)	*p*^b^ = 0.250
Glottal-to-noise excitation ratio	0.76 ± 0.21 (0.85)	0.75 ± 0.16 (0.77)	0.75 ± 0.18 (0.82)	*p*^b^ = 0.561

Notes: ^a^ Student *t* -test with equal variance. ^b^ Mann-Whitney test.

In total, 5% of the participants assessed had an altered fundamental frequency, 17.5% had altered jitter values, 30% had altered shimmer values, and 10% had altered GNE ratios ( Table 4 ). However, these values were not significantly different between the two groups.

**Table 4:** Statistics of the acoustic analysis by study group

	Study groups						
Acoustic analysis	Group I		Group II		Whole sample		*p* -value
	n	%	n	%	N	%	
**Fundamental frequency: male**							*p*^a^ = 1.000
Altered	1	10.0	–	–	1	5.0	
Normal	9	90.0	10	100.0	19	95.0	
**Fundamental frequency: female**							*p*^a^ = 1.000
Altered	–	–	1	10.0	1	5.0	
Normal	10	100.0	9	90.0	19	95.0	
**TOTAL**	**20**	**100.0**	**20**	**100.0**	**40**	**100.0**	
**Jitter**							
Altered	4	20.0	3	15.0	7	17.5	*p*^a^ = 1.000
Normal	16	80.0	17	85.0	33	82.5	
**Shimmer**							
Altered	6	30.0	6	30.0	12	30.0	*p*^b^ = 1.000
Normal	14	70.0	14	70.0	28	70.0	
**Glottal-to-noise excitation ratio**							
Altered	2	10.0	2	10.0	4	10.0	*p*^a^ = 1.000
Normal	18	90.0	18	90.0	36	90.0	
**TOTAL**	**20**	**100.0**	**20**	**100.0**	**40**	**100.0**	

Notes: ^a^ Fisher exact test. ^b^ Pearson Chi-squared test.

The assessment performed by the examiner with the greatest internal agreement, as analyzed through the kappa score, was used for the perceptual auditory analysis. These evaluators had scores for internal reliability ranging from good to perfect (0.667 to 1.00). The overall mean for each AVS parameter was used to classify the voices according to the degree of alteration. Table 5 shows that both the OSA and each of the individual parameters assessed had normal variability in voice quality. Although the values for pitch were within the normal range, they differed between the groups.

**Table 5:** Statistics of the perceptual auditory analysis by study group

	Study groups			
Variable	Group I (n = 20)	Group II (n = 20)	Whole sample (N = 40)	
	Mean ± standard deviation (median)	Mean ± standard deviation (median)	Mean ± standard deviation (median)	*p* -value
**Perceptual auditory parameters**				
Overall severity of alteration	13.15 ± 13.37 (12.00)	10.05 ± 14.15 (1.00)	11.60 ± 13.68 (9.50)	*p*^a^ = 0.464
Roughness	11.90 ± 13.01 (10.50)	9.85 ± 14.14 (0.00)	10.88 ± 13.45 (4.50)	*p*^a^ = 0.435
Breathiness	17.65 ± 19.00 (14.00)	11.25 ± 12.35 (7.00)	14.45 ± 16.15 (8.00)	*p*^a^ = 0.076
Tension	20.75 ± 3.37 (20.00)	15 ± 2.24 (12.20)	16.48 ± 13.5 (15.50)	*p*^a^ = 0.067
Pitch	12.75 ± 3.16 (12.00)	9.85 ± 1.84 (10.00)	11.30 ± 2.95 (11.00)	*p*^a^ = 0.002*
Loudness	1.40 ± 4.36 (0.00)	0.00 ± 0.00 (0.00)	0.70 ± 3.12 (0.00)	*p*^a^ = 0.152

Notes: *Significant difference at the level of 5.0%. ^a^ Mann-Whitney test.

Most of the older adults assessed presented a normal performance in the PPS (75%) and altered performance in the RGDT (57.5%) ( Table 6 ). Most of the older adults (65%) with altered performance in the RGDT were in group II.

**Table 6:** Results of the Pitch Pattern Sequence and Random Gap Detection Test

	Study groups						
Temporal auditory processing	Group I		Group II		Whole sample		*p* -value
	n	%	n	%	N	%	
TOTAL	20	100.0	20	100.0	40	100.0	
**Pitch Pattern Sequence**							*p*^a^ = 1.000
Altered	5	25.0	5	25.0	10	25.0	
Normal	15	75.0	15	75.0	30	75.0	
**Random Gap Detection Test**							*p*^a^ = 0.337
Altered	10	50.0	13	65.0	23	57.5	
Normal	10	50.0	7	35.0	17	42.5	

Note: ^a^ Pearson Chi-squared test.

Table 7 shows that the average percentage of correct responses in the PPS test among the older adults was of 78.63%. In the RGDT, the mean temporal resolution threshold for the older adults in group II was of 35.59 ms. In the group comparison, group II had a higher mean threshold than group I. However, there was no significant difference between the groups. A high variability in performance was noted for the RGDT. Table 7 also shows the results of the MPP test, with higher mean thresholds for target signal detection when it was presented with a shorter time gap. However, the mean thresholds did not differ between the groups.

**Table 7:** Mean values for performance in the temporal auditory processing assessment tests

	Study groups			
Variable	Group I (n = 20)	Group II (n = 20)	Whole sample (N = 40)	
	Mean ± standard deviation (median)	Mean ± standard deviation (median)	Mean ± standard deviation (median)	*p* -value
**Temporal auditory processing tests**				
Pitch Pattern Sequence	80.25 ± 19.16 (85.00)	77.00 ± 18.31 (77.50)	78.63 ± 18.57 (85.00)	*p*^b^ = 0.479
Random Gap Detection Test	34.23 ± 24.26 (24.00)	36.95 ± 34.08 (31.13)	35.59 ± 29.23 (25.50)	*p*^b^ = 0.946
Masking Period Pattern: Steady-high	73.70 ± 10.42 (72.63)	73.96 ± 10.27 (70.35)	73.83 ± 10.21 (70.97)	*p*^b^ = 0.871
Masking Period Pattern: Steady-low	67.35 ± 16.11 (67.83)	61.59 ± 20.24 (65.11)	64.47 ± 18.29 (65.11)	*p*^a^ = 0.326
Masking Period Pattern: 35 ms	74.47 ± 10.68 (74.50)	79.69 ± 9.73 (75.17)	77.08 ± 10.42 (75.06)	*p*^b^ = 0.194
Masking Period Pattern: 135 ms	72.80 ± 13.16 (73.33)	72.96 ± 13.56 (70.38)	72.88 ± 13.19 (72.44)	*p*^a^ = 0.971
Masking Period Pattern: 200 ms	76.54 ± 6.41 (74.16)	77.33 ± 8.52 (73.99)	76.93 ± 7.46 (74.05)	*p*^b^ = 0.957

Notes: ^a^ Student *t* -test with equal variance. ^b^ Mann-Whitney test.

Table 8 shows the comparison of voice analysis and auditory processing in terms of gender, without considering the presence or absence of vocal symptoms. Differences related to frequency, shimmer, OSA, and roughness (perceptual auditory analysis) were verified. As for the performance in the assessment of temporal auditory processing, no difference was observed regarding gender.

**Table 8:** Acoustic, perceptual auditory, and temporal auditory processing analyses by gender

	Gender		
Variable	Male (n = 20)	Female (n = 20)	
	Mean ± standard deviation (median)	Mean ± standard deviation (median)	*p* -value
**Acoustic analysis**			
Frequency	117.15 ± 15.19 (116.48)	177.45 ± 21.67 (179.29)	*p*^a^ ≤ 0.001*
Intensity	58.93 ± 6.16 (58.34)	57.16 ± 5.40 (58.30)	*p*^a^ = 0.339
Jitter	0.61 ± 0.99 (0.31)	0.49 ± 0.77 (0.23)	*p*^b^ = 0.120
Shimmer	7.05 ± 3.91 (6.14)	4.55 ± 3.69 (3.44)	*p*^b^ = 0.009*
Glottal-to-noise excitation ratio	0.71 ± 0.19 (0.70)	0.79 ± 0.16 (0.85)	*p*^b^ = 0.123
**Perceptual auditory analysis**			
Overall severity of alteration	18.30 ± 14.25 (18.00)	4.90 ± 9.30 (0.00)	*p*^b^ < 0.001*
Roughness	16.75 ± 14.44 (17.00)	5.00 ± 9.51 (0.00)	*p*^b^ = 0.003*
Breathiness	14.95 ± 14.16 (12.00)	13.95 ± 18.28 (0.00)	*p*^b^ = 0.473
Tension	0.15 ± 0.67 (0.00)	0.00 ± 0.00 (0.00)	*p*^b^ = 0.317
Pitch	11.45 ± 2.06 (11.00)	11.15 ± 3.67 (10.00)	*p*^b^ = 0.263
Loudness	1.40 ± 4.36 (0.00)	0.00 ± 0.00 (0.00)	*p*^b^ = 0.152
**Temporal auditory processing tests**			
Pitch Pattern Sequence	80.75 ± 16.49 (85.00)	76.50 ± 20.65 (82.50)	*p*^b^ = 0.683
Random Gap Detection Test	33.33 ± 33.19 (23.75)	37.85 ± 25.33 (36.88)	*p*^b^ = 0.372
Masking Period Pattern: Steady-high	73.33 ± 9.83 (72.55)	74.33 ± 10.82 (70.13)	*p*^b^ = 0.665
Masking Period Pattern: Steady-low	63.34 ± 17.22 (63.66)	65.60 ± 19.68 (65.33)	*p*^a^ = 0.701
Masking Period Pattern: 35 ms	75.40 ± 8.38 (75.00)	78.77 ± 12.12 (76.05)	*p*^c^ = 0.314
Masking Period Pattern: 135 ms	72.15 ± 12.27 (71.08)	73.61 ± 14.33 (73.11)	*p*^a^ = 0.732
Masking Period Pattern: 200 ms	76.77 ± 7.12 (74.22)	77.10 ± 7.96 (73.44)	*p*^b^ = 0.756

Notes: *Significant difference at the level of 5.0%. ^a^ Student *t* -test with equal variance. ^b^ Mann-Whitney test. ^c^ Student *t* -test with unequal variance.

In the analysis of the correlation involving the participants' age and auditory processing and voice, the results indicate an indirect relationship between age and loudness. On the other hand, the relationship between age and the mean thresholds on the MPP test was direct, as all the correlations regarding these variables were positive ( Table 9 ).

**Table 9:** Spearman correlation regarding age and the acoustic, the perceptual auditory, and the temporal auditory processing assessments

Variable	Age
**Acoustic parameters**	
Fundamental frequency (male)	−0.247 (0.294)
Fundamental frequency (female)	0.030 (0.899)
Intensity	0.115 (0.481)
Jitter	0.309 (0.052)
Shimmer	0.331 (0.037)
Glottal-to-noise excitation ratio	−0.260 (0.106)
**Perceptual auditory parameters**	
Overall severity of alteration	0.151 (0.35)
Roughness	0.123 (0.45)
Breathiness	0.292 (0.06)
Tension	0.118 (0.46)
Pitch	0.249 (0.12)
Loudness	−0.309 (0.050)*
**Temporal auditory processing tests**	
Pitch Pattern Sequence	0.086 (0.598)
Random Gap Detection Test	0.150 (0.354)
Masking Period Pattern: Steady-high	0.516 (0.001)*
Masking Period Pattern: Steady-low	0.501 (0.001)*
Masking Period Pattern: 35 ms	0.384 (0.014)*
Masking Period Pattern: 135 ms	0.473 (0.002)*
Masking Period Pattern: 200 ms	0.522 (0.001)*

Note: *Statistically different from zero.

In the correlation analysis between auditory processing and perceptual auditory voice parameters, a positive correlation was identified regarding the temporal resolution skill, the OSA, and voice roughness ( Table 10 ). However, the overall voice grade and roughness values were within the normal range according to the cutoff score on the VAS.

**Table 10:** Spearman correlation regarding the perceptual auditory analysis and the temporal auditory processing

	Perceptual auditory parameters					
Temporal auditory processing tests	Overall severity of alteration	Roughness	Breathiness	Tension	Pitch	Loudness
**Pitch Pattern Sequence**	0.134 (0.409)	0.265 (0.099)	0.236 (0.143)	−0.004 (0.980)	0.011 (0.945)	−0.030 (0.852)
**Random Gap Detection Test**	0.420 (0.007)*	0.316 (0.047)*	0.233 (0.148)	−0.243 (0.131)	−0.071 (0.664)	0.220 (0.172)
**Masking Period Pattern: Steady-high**	−0.144 (0.377)	−0.100 (0.539)	−0.028 (0.862)	−0.049 (0.766)	0.139 (0.391)	0.278 (0.082)
**Masking Period Pattern: Steady-low**	−0.254 (0.114)	−0.243 (0.131)	−0.090 (0.580)	0.049 (0.766)	0.033 (0.839)	0.219 (0.174)
**Masking Period Pattern: 35 ms**	−0.180 (0.267)	−0.064 (0.695)	0.022 (0.891)	0.132 (0.418)	−0.062 (0.702)	0.180 (0.268)
**Masking Period Pattern: 135 ms**	−0.081 (0.619)	−0.232 (0.149)	0.061 (0.708)	−0.090 (0.580)	0.117 (0.471)	0.257 (0.109)
**Masking Period Pattern: 200 ms**	−0.039 (0.810)	−0.110 (0.499)	0.125 (0.441)	0.062 (0.702)	0.067 (0.681)	0.284 (0.075)

Note: *Statistically different from zero.

No correlation was identified between the auditory processing assessment and the acoustic analysis of the voice, as shown in Table 11 .

**Table 11:** Spearman correlation between acoustic analysis and temporal auditory processing

Temporal auditory processing	Frequency		Intensity	Jitter	Shimmer	Glottal-to-noise excitation ratio
	Male	Female				
**Pitch Pattern Sequence**	−0.244 (0.299)	−0.174 (0.463)	−0.270 (0.092)	0.163 (0.316)	0.232 (0.150)	0.131 (0.420)
**Random Gap Detection Test**	−0.261 (0.266)	0.212 (0.369)	−0.247 (0.125)	−0.019 (0.908)	−0.163 (0.316)	−0.017 (0.917)
**Masking Period Pattern: Steady-high**	−0.319 (0.171)	0.317 (0.173)	0.160 (0.323)	−0.023 (0.890)	0.067 (0.679)	−0.273 (0.088)
**Masking Period Pattern: Steady-low**	−0.020 (0.935)	0.298 (0.202)	0.104 (0.525)	−0.064 (0.695)	−0.003 (0.983)	−0.147 (0.367)
**Masking Period Pattern: 35 ms**	−0.224 (0.342)	−0.036 (0.880)	0.229 (0.154)	−0.028 (0.864)	−0.108 (0.508)	−0.204 (0.207)
**Masking Period Pattern: 135 ms**	0.000 (1.000)	0.165 (0.486)	0.077 (0.638)	0.083 (0.610)	−0.028 (0.865)	−0.169 (0.299)
**Masking Period Pattern: 200 ms**	−0.257 (0.274)	0.167 (0.482)	0.209 (0.195)	0.098 (0.547)	0.063 (0.700)	−0.261 (0.103)

## Discussion

Voice production depends on the coordination of subsystems of the vocal tract, which are affected in different ways with advancing age. Voice symptoms can also be related to diminished respiratory support, resonance alterations, and reduced orofacial musculature tonicity. 9 19 20 21 Moreover, there is evidence that hearing is associated with voice production. 22 23

Auditory disorders can directly affect the feedback mechanism involved in producing voice, so that control of the immediate and suprasegmental speech components, such as the fundamental frequency, jitter, and shimmer, is impaired. 24 One example of this relationship concerns the significant differences in the parameters associated with the characteristics of voice in individuals with hearing loss. 25

Among indviduals aged ≥ 60 years, the prevalence of complaints regarding the occurrence of voice symptoms ranges from 4.8% to 29.1%. 26 Some frequent complaints are poor voice projection, reduced vocal resistance, and, consequently, reduced phonation time, fatigue or shortness of breath, and roughness. 27 28 In the present study, the older adults in group I presented more voice symptoms according to the VoiSS than those in group II, except for complaints of deep voice.

Of the eight complaints reported in the present study, only three (throat clearing, fatigue, and weak voice) differed between participants with and without voice symptoms. As aforementioned, voice complaints are common in the elderly due to the natural aging of the subsystems involved in phonation. 9 Hence, the scores below the cutoff the VoiSS may not eliminate the occurrence of voice complaints in this population. Furthermore, the complaints that differed between the groups of older adults can be related to individual susceptibilities and characteristics, such as general health, physical health, life habits, and so forth. 29 30

Clearing the throat, roughness, and coughing are voice symptoms often associated with esophageal disorders such as the passive return of gastric content to the esophagus, which can occur in any individual due to physiological or pathological circumstances (gastroesophageal reflux disease), but it is common in older adults. 31 32 Moreover, the self-reported throat clearing can be related to the sensation that causes the constant need to try and clear the throat when swallowing. 33

Some voice symptoms in older adults can also be associated with changes in the respiratory system. Fatigue and weak voice are complaints mainly related to the reduction in respiratory support. 28 34 For phonation to occur, the aerodynamic and muscular forces must be balanced, ensuring enough transglottal airflow and adequate vibration of the vocal folds. 35 36

Concerning the self-evaluation of voice, most of the participants in group I had a negative perception, which agrees with the greater occurrence of voice complaints found in this group. Nonetheless, considering the totality of older adults participating in the present study, we noted that most of them had evaluated their voices positively, classifying them as either good, very good or excellent. This suggests that older adults are not always able to aurally distinguish the alteration in their vocal quality. However, they seem to notice the impact such changes have, as they presented higher scores on the physical and functional domains than on the emotional domain of the VoiSS, as already described by other authors. 37

In the self-evaluation of voice, the perception of a deep or shrill voice can be related to both laryngeal structural alterations and hormonal ones, leading to changes in the fundamental frequency of the voice, regardless of vocal alteration. A tendency towards deep voice complaints has been observed to be more frequent among women, and shrill voice complaints are more prevalent among men. 29 This is due to the fact that women present significant hormonal changes after menopause, which, as a result, can cause the thickening of the mucous membrane of the vocal fold, thus reducing the frequency of the voice. In men, the fundamental frequency tends to increase as a consequence of atrophy in the musculature of the vocal folds, reducing the amount of vibrating mass during phonation. 38 39

Regarding the acoustic analysis of the voice, according to the VoiSS, groups I and II did not differ regarding the parameters assessed. Jitter and shimmer are indicators of disturbance in the mucous wave of the vocal folds; jitter indicates the variability in the fundamental frequency cycle-to-cycle, and shimmer, the variability of the wave amplitude cycle-to-cycle. Both measurements can vary and increase in older adults in general, regardless of the voice alteration identified, because the variations in disturbance of the mucous wave depend on structural and functional laryngeal modifications that naturally take place with advancing age, as do the loss of muscle mass, decrease in muscle tone, and atrophy of the vocal muscle. 40 Likewise, the literature indicates that the proportion of GNE of the mucous wave is influenced by vocal aging, and it is also associated with variations in amplitude and frequency. 41

Groups I and II did not differ concerning the parameters of the perceptual auditory assessment, except for pitch, which is correlated with the fundamental frequency of the voice. Changes in this parameter occur naturally with advancing age, as it is associated with different anatomophysiological changes, regardless of the occurrence of vocal symptoms. 38 39

Concerning the acoustic parameter of intensity, the mean of the values found in the present study was lower in comparison with the mean found in the literature. 34 41 It is known that different mechanisms are involved in the control of loudness, the psychoacoustic sensation of the intensity or sonority, both in young individuals and in older adults. The changes in vocal volume or projection are mainly related to reduced respiratory support. 28 34 As it is known, reduced airflow with decreased subglottal air pressure occurs with advancing age, due to the loss of muscle mass, strength, and/or reduced activity. 42 In addition, it is important to emphasize the influence of the conditions under which the voice is recorded conditions to ensure the reliability of the sample and of the analysis. Therefore, in the present study, the positioning of the microphone in relation to the mouth was controlled.

There is evidence that impaired auditory perception of the intensity or sonority of the voice can be equally associated with loudness alteration in older adults. 43 It has emphasized that the aging of auditory system can interfere with speech discrimination and affect auditory temporal perception.

Among the parameters assessed in the perceptual auditory voice analysis, normal variability in vocal quality or absence of deviation were identified, though a moderate degree of alteration in vocal quality had been previously verified 44 in institutionalized older adults. However, the older adults evaluated at the institution had a higher average age (75.7 years), which can explain the difference. 44 It is important to comment that the sample of the presentis study was composed of active older adults, who seek group activities and health practices in the place where the research was conducted. However, in the voice assessment of older women who perform aerobic activities, the perceptual auditory assessment showed a predominance of unaltered voices, and when there was a change in any parameter, it was mostly discrete. 30

The gender differences regarding shimmer, the OSA, and roughness found in the study are in line with those reported in the literature. 45 Just as in women, hormonal changes can also occur in men, such as the decrease in estradiol. Differences in the shimmer values can be related to these changes and to the atrophy or arching of the vocal folds, which occur more often in men. 45 Roughness, in turn, is related to phonatory irregularity, which is also more common in men, whereas breathiness is more expected to happen in women, due to glottal proportion and laryngeal configuration. 34 39

As for the temporal processing skills, they are necessary for the resolution of prosodic details. It is in the temporal auditory processing that the temporal cues for the discrimination of speech and vocalization are used. The prosody, the intonation, or the rhythm characteristics depend on the same neural systems as the auditory perception of tones, as in the order of the musical notes. 46

Most of the subjects in the current study presented a normal performance in the temporal ordering skill, as assessed through the PPS, and an altered performance in the temporal resolution skill, assessed through the RGDT.

A study 47 reported evidence of a significant difference in temporal resolution between men and women. The authors 47 propose that the male central auditory nervous system responds differently to non-verbal stimuli. However, an expressive difference was observed in the distribution between genders in the studied population. 47 Regarding this relationship, as in other publications in the literature, 12 48 the present study did not observe gender differences in terms of temporal ordering and resolution ability.

Auditory temporal resolution is the skill of detecting changes in the sound stimuli in relation to time, such as when it is necessary to perceive the occurrence of two stimuli instead of one. The inter-stimuli interval detection threshold, the minimum interval in which the individual was able to consistently identify the occurrence of two stimuli, was obtained via the RGDT.

The impairment in temporal resolution ability found in the studied population may be related to aspects concerning cognition and attention, as well as to the reduced speed of auditory information processing in the elderly. 47 It is also believed that the altered performance in the RGDT may be related to the instruction of the test. It is important to consider that, despite the performance in the training with the expanded version of the test (with longer intervals between the sound signals, which, therefore are easier to identify), there was high variability in the test performance in the sample of te present study, which was also observed in another study. 49

The MPP test showed that the mean thresholds for the different stimulus positions in relation to the presentation time in the presence of masking were close to the mean identification thresholds for the baseline. As in another recent study, 50 even the lowest thresholds for signal detection (presented 35ms after the displacement of the mask modulation) were higher in relation to the baseline (Steady-low). It has been shown 50 that the shorter the time interval between the presentation of masking noise and the presentation of the target signal, the greater the chance of an elevated threshold, due to the permanence of masking in the elderly population. It is noteworthy that the authors found mean thresholds for the speech signal below the mean thresholds found for the target signal in the present study.

The mean thresholds found in the present study suggest that the signal detection cues are greater or more easily perceived in a constant-masking situation than in an oscillating one, indicating the permanence of an effective non-simultaneous masking. The present study indicates that older adults are more susceptible to the masking effect, which is in line with other studies in the literature. 51 52 53

In addition, the positive and significant correlation between the detection thresholds in the different masking conditions and the subject's age shows that the older subjects perform worse in the presence of masking noise. This would also reinforce the effects of the simultaneous temporal masking in this population. It is believed that such permanence of the temporal masking leads to the reduced signal-to-noise harnessing skill also during the minimal periods of masking; hence, the acoustic cues important for speech end up masked, so that both the speech comprehension and the auditory feedback that regulates vocal production are impaired. 54 55 56

The high thresholds in the temporal masking assessment suggest that, with advancing age, the acoustic cues are perceived with more difficulty, even when the noise is modulated due to the permanence of the masking. Still on the mean thresholds found, the time taken to conduct the assessment may have an influence on the MPP results, since sustained attention is required, which is tiring when other tests are applied in the same session.

With advancing age, changes in the auditory nerve fibers lead to a decreased speed in the poststimulation spontaneous return. As suggested by other authors, 54 the elevated auditory thresholds for the identification of the target signal in the MPP test can be related to these and other physiological aspects of aging.

Considering that auditory monitoring enables better control of the tone, volume, and intelligibility of the voice, it is believed that, when impaired, negative consequences can emerge, such as inadequate vocal adjustments, and loss or damage to the vocal quality or comfort. It is understood that voice production involves a sensorimotor integration in which there is fine motor planning for the phonation and articulation. 57 The voice produced is perceived by the subject and submitted to adjustments and corrections in case discrepancies are identified between the quality of the production and the speaker's intention. These adjustments are organized in mental representations and stored for the better planning of future voice and speech emissions. This whole mechanism involves the perception of the tone (auditory pitch mapping) and the sensorimotor integration for voice production, which is referred to as auditory motor mapping or sensorimotor loop. 57

The permanence of the auditory masking and the alteration in temporal resolution in older adults are auditory conditions that compromise the harnessing of the sound signal produced and, consequently, the auditory feedback. In this regard, the correlation found involving the temporal resolution skill and the parameters of roughness and OSA is highlighted. It is believed that impaired auditory feedback can lead to phonatory adjustment with effort, which, in turn, can lead to vibratory irregularity, with roughness characteristics. Concerning the perceptual auditory measures, it has been made evident that roughness is one of the most altered parameters in aging, usually in slight to moderate degrees, 27 58 and it is related to phonatory irregularity or muscular activity.

For the adequate motor planning of phonation to take place, and for it to meet the speaker's needs, the auditory representation of the vocal performance must be correctly mapped and occur without loss concerning time. 50 59 60

The study of the correlation between the perception of auditory difficulties and vocal symptoms was relevant in the present research. The question is raised as to how much effort in voice production would be related to the effort in performing auditory tasks; hence, the need for further evidence on the relationship studied is presented.

Reinforcing the evidence on this relationship enables the advent of treatment alternatives that are better adjusted to the needs of this population, with approaches aimed at their auditory and communicative skills.

## Conclusion

In older adults, there is damage to the processing of temporal auditory information at central levels, which was correlated with vocal symptoms. This relationship can be explained by inadequate vocal adjustments. It is important to highlight that the correlation does not suggest causality. However, the findings of the present study support the importance of conducting further research and analyses, considering the extension of the variables involved.
